# Reply to: “Global conservation of phylogenetic diversity captures more than just functional diversity”

**DOI:** 10.1038/s41467-019-08603-5

**Published:** 2019-02-20

**Authors:** Florent Mazel, Matthew W. Pennell, Marc W. Cadotte, Sandra Diaz, Giulio Valentino Dalla Riva, Richard Grenyer, Fabien Leprieur, Arne O. Mooers, David Mouillot, Caroline M. Tucker, William D. Pearse

**Affiliations:** 1 Department of Biological Sciences, Simon Fraser University, Burnaby, V5A 1S6 BC Canada; 2 Department of Botany, University of British Columbia, Vancouver, BC V6T 1Z4 Canada; 3 Biodiversity Research Centre, University of British Columbia, Vancouver, BC V6T 1Z4 Canada; 4 Department of Zoology, University of British Columbia, Vancouver, BC V6T 1Z4 Canada; 5 Biological Sciences, University of Toronto-Scarborough, Scarborough, M1C 1A4 Canada; 6 Ecology and Evolutionary Biology, University of Toronto, Toronto, M5S 3B2 ON Canada; 7 Instituto Multidisciplinario de Biología Vegetal, CONICET and FECFyN, Universidad Nacional de Córdoba, Casilla de Correo 495, 5000 Córdoba, Argentina; 8 School of Mathematics and Statistics, University of Canterbury, Christchurch, 8140 New Zealand; 9 School of Geography and the Environment, University of Oxford, Oxford, OX1 3QY UK; 10 Marine Biodiversity, Exploitation, and Conservation (MARBEC), UMR 9190, Université de Montpellier, Montpellier, 34095 France; 11 Australian Research Council Centre of Excellence for Coral Reef Studies, James Cook University, Townsville, 4811 QLD Australia; 12 Department of Biology, University of North Carolina-Chapel Hill, Chapel Hill, 27599-3280 NC USA; 13 Ecology Center and Department of Biology, Utah State University, Logan, 84322 UT USA

**Replying To** N. R. Owen et al. *Nature Communications* 10.1038/s41467-019-08600-8 (2019)

Academic biologists have long advocated for conserving phylogenetic diversity (PD), often (but not exclusively) on the basis that PD is a useful proxy for “feature diversity”, defined as the variety of forms and functions represented in set of organisms (see below for an extended discussion of this definition)^1–4^. In a recent paper^5^, we assess the extent to which this proxy (which we coined the “phylogenetic gambit”) holds in three empirical datasets (terrestrial mammals, birds, and tropical marine fishes) when using functional traits and functional diversity (FD) to operationalize feature diversity. Owen et al.^6^ offer a criticism of our methods for quantifying feature diversity with FD and disagree with our conclusions. We are grateful that Owen et al.^6^ have engaged thoughtfully with our work, but we believe there are more points of agreement than Owen et al.^6^ imply.

The broad conclusion of our empirical study, which incorporated >15,000 vertebrate species and a limited number of traits, was that the phylogenetic gambit holds for ecologically relevant traits: maximizing PD results in an average gain of 18% of FD relative to random choice^5^. However, as clear from the title of our paper, PD’s reliability as a surrogate for FD as we measured it was also surprisingly weak: the 18% gain masks the fact that, in 1/3 of the comparisons, maximizing PD resulted in less FD than a random choice. This finding is in line with previous assessments^7, 8^. Importantly, though, we did not argue that we “need to abandon the use of PD in conservation”^6^, and indeed we opined that doing so would be a “dramatic decision”[5]. We called for further tests of the phylogenetic gambit in other taxonomic groups, at a variety of spatial scales, and across many types of traits.

Owen et al.^6^ offer three linked arguments: (1) FD is an inadequate measure of feature diversity in conservation; (2) our tests ignore spatial scale in conservation prioritization; and (3) our work is not supported by other tests of the phylogenetic gambit. We touch on each of these below.

1. In the phylogenetic gambit, PD is a potential proxy for feature diversity. Feature diversity is an important concept, but as Faith^1^ also noted, one that is “difficult to estimate directly”. We provided one potential approach for operationalizing the concept, by measuring FD as is done in ecology. Importantly, we focused on ecological traits (body mass, diet, foraging strata, and foraging time) because we felt these are often related to conservation outputs of interest to humans (particularly via local ecosystem services). We emphasized that “many other potential[ly] valuable traits are not captured by our measure of FD”^5^, but we do see how our phrase “diversity of form and function can be measured as FD” might be taken to include an implicit “exclusively.” That was not our intent. Critically, there is some reason to believe that other measures of feature diversity (e.g., some integrated measures of total variation over many phenotypic axes) may covary differently with PD. This means that an essential next step will be to determine how trait choice, trait number and trait measurement impact the phylogenetic gambit. Critically, though, future operationalization of feature diversity must be coupled with tests of the potential benefits to people of what is being measured (see our point (3)). Thus, we fully agree with Owen et al.^6^ that our analysis is but one test of the phylogenetic gambit, and we hope this exchange spurs other approaches for measuring feature diversity.
2. Owen et al.^6^ correctly highlight that our geographic (assemblage-based) prioritization analyses do not consider how PD captures FD at a global scale. Their empirical example of such global vs. local considerations for corals is useful. We did not consider corals but coral fishes in our paper, though they are mentioned in the newspaper article they quote. Our assemblage-based analysis, however, was only one half of our study: the other was a global analysis of the phylogenetic gambit in which we found results consistent with those of our assemblage-based analyses (surrogate estimates were 15% and 21%, respectively). Thus, our results are likely robust to the concerns Owen et al.^6^ raise about our data and our measure of FD.
3. While few would disagree with Owen et al.^6^ that their snake and marsupial anecdotes are exciting examples of the potential benefits of using phylogeny for prioritization and conservation, scientific hypotheses must be confronted with data, not anecdotes. Owen et al.^6^ cite a recent book chapter^9^ (ref. four) as a test of the phylogenetic gambit; however, we found only a single-empirical study referenced there. That study^10^ surveyed and found phylogenetic signal for various traits (although the pattern was not universal). We agree that phylogenetic signal is widespread and common^11^, but we have already shown^8^ that the presence of phylogenetic signal does not guarantee that the phylogenetic gambit holds as we tested it. This counter-intuitive result may be important as we consider further evidence.

Theory is foundational to scientific advance, but it is only a first step. We suggest that the only way to move this field forward is to empirically assess statistical support for all linkages between PD and benefits to people (see Forest et al.^12^ for one example). The issue is not academic: managers and policy makers grappling with urgent conservation decisions require evidence. We welcome further tests that link other metrics to other traits and to other explicit benefits.
